# Correction: The longitudinal association between patient empowerment and patient-reported outcomes: What is the direction of effect?

**DOI:** 10.1371/journal.pone.0314216

**Published:** 2024-11-15

**Authors:** Mariela Acuña Mora, Carina Sparud-Lundin, Eva Fernlund, Shalan Fadl, Kalliopi Kazamia, Annika Rydberg, Åsa Burström, Katarina Hanseus, Philip Moons, Ewa-Lena Bratt

The fifth author’s first and last name are incorrectly switched. The correct name is: Kalliopi Kazamia. The correct citation is: Acuña Mora M, Sparud-Lundin C, Fernlund E, Fadl S, Kazamia K, Rydberg A, et al. (2022) The longitudinal association between patient empowerment and patient-reported outcomes: What is the direction of effect? PLOS ONE 17(11): e0277267. https://doi.org/10.1371/journal.pone.0277267.
