# Supplementary material for: The longitudinal association between patient empowerment and patient-reported outcomes: What is the direction of effect?
Source: PLoS One. 2022 Nov 10;17(11):e0277267. doi: 10.1371/journal.pone.0277267 (PMC9648754; doi:10.1371/journal.pone.0277267)
Supplement: S1 File — (DOCX) [file pone.0277267.s001.docx]

| **Model fit indices from the cross-lagged panel models and the random intercept cross-lagged panel models** | | | | | | | |
| --- | --- | --- | --- | --- | --- | --- | --- |
|  | **X^2^ (*df*)** | **CFI** | **RMSEA** | **SRMR** | **∆CFI** | **∆RMSEA** | **∆SRMR** |
| ***Patient empowerment and quality of life*** | | | | | | |  |
| CLPM model | 22.992 (4) | 0.920 | 0.185 | 0.053 |  |  |  |
| RI-CLPM model | 5.464 (1) | 0.981 | 0.180 | 0.037 | 0.061 | 0.005 | 0.016 |
| ***Patient empowerment and patient-reported health*** | | | | | | |  |
| CLPM model | 35.705 (4) | 0.998 | 0.061 | 0.028 |  |  |  |
| RI-CLPM model | 1.519 | 0.848 | 0.240 | 0.087 | 0.15 | 0.179 | 0.059 |
| ***Patient empowerment and communication*** | | | | | | |  |
| CLPM model | 18.213 (4) | 0.932 | 0.160 | 0.062 |  |  |  |
| RI-CLPM model | 1.263 (1) | 0.999 | 0.044 | 0.023 | 0.067 | 0.116 | 0.039 |
| ***Patient empowerment and transition readiness*** | | | | | | |  |
| CLPM model | 21.773 (4) | 0.901 | 0.179 | 0.056 |  |  |  |
| RI-CLPM model | 0.412 (1) | 1.000 | 0.000 | 0.012 | 0.099 | 0.179 | 0.044 |
| CLPM: cross-lagged panel model; RI-CLPM: random intercept cross-lagged panel model; CFI: comparative fit index; RMSEA: Root mean square error of approximation; SRMR: Standardized root mean square residual (SRMR)  Models were considered to have acceptable fit if CFI > 0.90, RMSEA and SRMR values < 0.08. | | | | | | | |
