# Supplementary material for: The longitudinal association between patient empowerment and patient-reported outcomes: What is the direction of effect?
Source: PLoS One. 2022 Nov 10;17(11):e0277267. doi: 10.1371/journal.pone.0277267 (PMC9648754; doi:10.1371/journal.pone.0277267)
Supplement: Attachment — Submitted filename: Response to reviewers Rev2.docx [file pone.0277267.s003.docx]

Dr. Thomas Philips (Staff Editor)

PLOS ONE

Submission of manuscript

Dear Dr. Philips,

Please find attached the revised version of the manuscript entitled **“The longitudinal association between patient empowerment and patient-reported outcomes: what is the direction of effect?”.** We would like to resubmit this manuscript for consideration by PLOS ONE.

We thank you for the opportunity to revise and resubmit the manuscript. Furthermore, we would like to thank the reviewer for the constructive comment. Enclosed is a letter summarizing the rebuttal on the comment from the reviewer. Corrections and additions made in the manuscript have been marked using the “track changes” option of MS Word.

We hope that we have sufficiently addressed your comments. Thank you for considering this manuscript for publication in the PLOS ONE.

On behalf of all co-authors,

Mariela Acuña Mora.

**Reviewer #1**

**The authors fully addressed most of the comments. There is one issue on MCID (Page 10, line 172) that require more clarification from the authors. Specifically, they use the standard error (SE) less than 1 to reflect whether the scale is showing MCID. Since SE are sample size dependent, their values might not reflect clinical importance of the scale measurement results. For example, a very large study done in a population with very low QoL may yield a SE less than 1, and using SE one will erroneously conclude that the QoL in this population is high. In fact, SE in such a population is guaranteed to fall below 1 as long as you keep on increasing the sample size. On the other hand, a modest size study done on a population with high QoL (on average) may result in SE greater than 1. Perhaps you should consider using SD instead, as it reflects variability between subject measurements (on average) rather than the variability on a sample statistic.**

As the reviewer points out, the standard error (SE) is sample size dependent. This means that as the sample size increases, the smaller the SE becomes and vice versa. This characteristic of the SE can have an impact on the way the MCID is calculated and hence, interpreted. While other distribution-based methods to calculate the MCID are available, for instance methods that use the standard deviation (SD), there are also limitations when following this approach. One of the most relevant limitations of using the SD is that the MCID calculation may be sample dependent, and thus the calculation is less generalizable [1, 2]. MCIDs that rely on the SE allow for a higher generalizability of the scores [1, 2]. Another benefit of using the SEM is that it is expressed in the original metric of a measure, which facilitates the interpretation of the results [2].

Despite its limitations, the calculation of the MCID using the SE is frequently used in studies [2, 3, 4] and the available evidence suggests that this approach is reliable for identifying MCID [2]. It is also worth noting that the MCID varies based on the sample and the context. Therefore, it should not be accepted as a universal fact [1]. The interpretation of the MCID should be made with caution, while keeping in mind the limitations of the selected approach. One solution could be to use different approaches when reporting the MCID so the reader can make a comparison of the values based on different methods [1, 5]. We have reflected on this option but decided not do so, because it would bring the discussion on the MCID beyond perspective, and it would distract the reader from the main focus of the article, being the direction of effects. Thank you for your understanding.

**References**

1. Sedaghat AR. Understanding the Minimal Clinically Important Difference (MCID) of Patient-Reported Outcome Measures. Otolaryngology–Head and Neck Surgery. 2019;161(4):551-60.

2. Wyrwich, K.W., Tierney, W.M. & Wolinsky, F.D. Further Evidence Supporting an SEM-Based Criterion for Identifying Meaningful Intra-Individual Changes in Health-Related Quality of Life. J Clin Epidemiol. 1999; 52(9), pp.861–873.

3. Ousmen, A., Touraine, C., Deliu, N. et al. Distribution- and anchor-based methods to determine the minimally important difference on patient-reported outcome questionnaires in oncology: a structured review. Health Qual Life Outcomes. 2018; 16(228). https://doi.org/10.1186/s12955-018-1055-z

4. Mouelhi, Y. et al. How is the minimal clinically important difference established in health-related quality of life instruments? Review of anchors and methods. Health Qual Life Outcomes. 2020; 18(1), p.136.

5. Crosby RD, Kolotkin RL, Williams GR. Defining clinically meaningful change in health-related quality of life. J Clin Epidemiol. 2003;56(5):395-407.
